# Prevalence, Antimicrobial Susceptibility Pattern of Bacterial Isolates, and Associated Factors of Urinary Tract Infections among HIV‐Positive Patients at Hiwot Fana Specialized University Hospital, Eastern Ethiopia

**DOI:** 10.1155/2019/6780354

**Published:** 2019-02-06

**Authors:** Dadi Marami, Senthilkumar Balakrishnan, Berhanu Seyoum

**Affiliations:** ^1^Department of Medical Laboratory Sciences, College of Health and Medical Sciences, Haramaya University, P.O. Box: 235, Harar, Ethiopia; ^2^Department of Medical Microbiology, College of Health and Medical Sciences, Haramaya University, P.O. Box: 235, Harar, Ethiopia

## Abstract

Urinary tract infection remains a major public health problem in developing countries, where there are limited health-care services. Its prevalence is fueled by human immunodeficiency virus (HIV) infection. The emergence of antimicrobial resistance is now widespread and poses a serious clinical threat. This study investigated the prevalence, antimicrobial susceptibility pattern of bacterial isolates, and associated factors of urinary tract infections among HIV-positive adult patients. A cross-sectional study was conducted among 350 randomly selected HIV-positive patients at Hiwot Fana Specialized University Hospital from February to March 2016. Data were collected using a structured questionnaire. Clean-catch midstream urine samples were collected aseptically and examined using the recommended culture methods. Antimicrobial susceptibility testing was performed using the Kirby–Bauer disk diffusion technique. Data were analyzed using Statistical Package for the Social Sciences version 21.0. The logistic regression models were used to explore the predictors of the outcome. A *p* value < 0.05 was considered statistically significant. The overall prevalence of urinary tract infection was 18% (95% CI: 15.34–22.63). Individuals with age 35–44 years (Adjusted odds ratio (AOR): 4.07; 95% CI: 1.09, 5.10), income less than 46.7 USD (AOR: 2.76; 95% CI: 1.15, 6.07), and a CD4^+^ count less than 200 cells/mm^3^ (AOR: 2.07; 95% CI: 1.15, 3.73) had higher odds of UTI. *Escherichia coli* (38.1%), *Klebsiella pneumoniae* (23.8%), and *Staphylococcus aureus* (11.1%) were the predominant causes of urinary tract infection. *E. coli* was resistant to ampicillin (95.8%), ceftazidime (95.8%), cotrimoxazole (95.8%), amoxicillin (91.7%), ceftriaxone (87.5%), and tetracycline (87.2%). Multidrug resistance was observed in 46% of the isolates. The prevalence of urinary tract infection in this study was high compared to the previous reports in Ethiopia. Age 35–44 years, income less than 46.7 USD, and a CD4^+^ count < 200 cells/mm^3^ increase the odds of urinary tract infection. The most common isolates were *E. coli*, *K. pneumoniae*, and *S. aureus*. Almost half of the isolates were multidrug resistant. Actions to help mitigate the further spread of resistance are urgently needed in the study area.

## 1. Introduction

Urinary tract infection (UTI) is caused by the bacterial invasion and multiplication in the organs of the urinary tract system [1]. The frequency of UTI is gradually increasing amongst HIV-infected patients as an opportunistic infection. This is due to the unique pathogenesis of the virus, which decreases the CD4^+^ cells, and as such, the individual's immune system can no longer fight against invading commensal organisms [2–4]. *E. coli*, *Proteus* spp., *Klebsiella* spp., *Pseudomonas aeruginosa*, *Enterococcus* spp., and *S. aureus* are the most causative agent of UTI in people living with HIV [5, 6].

The health consequences of UTI among HIV-infected patients can be grave, resulting in acute and chronic kidney diseases [7], infertility, cancer, sepsis, and neurologic complication, which lead to urinary stasis [8, 9]. Some of the patients may substantially suffer from financial burden not only because of the recurrence of UTI but also due to the use of expensive antimicrobials, longer duration hospitalization, adverse drug effects, and unsatisfactory therapeutic options [7, 10].

The emergence of antimicrobial-resistant bacterial strains that poses a continued challenge to treat and control the spread of infections is another concern [10]. The problem is particularly immense in developing countries such as Ethiopia that do not have quality laboratory facilities to isolate pathogens and determine their antimicrobial susceptibility pattern, but with high fake drugs in circulation, misuse of antimicrobials by health-care providers, unskilled practitioners, and patients are common [2, 11].

In Ethiopia, limited studies are available regarding the extent of UTI and antimicrobial susceptibility profile in HIV-positive patients [2, 12]. This study investigated the prevalence, antimicrobial susceptibility pattern, and associated factors of bacterial UTI among HIV-positive adult patients at Hiwot Fana Specialized University Hospital, Eastern Ethiopia.

## 2. Materials and Methods

### 2.1. Study Setting

A hospital-based cross-sectional study was conducted at Hiwot Fana Specialized University Hospital, Harar, Ethiopia, from February to March 2016. The hospital is located at Harar, which is the capital city of the Harari Regional State, Ethiopia. The prevalence of HIV in the region was 3.2% (2.6% males and 3.8% females). Currently, Hiwot Fana Specialized University Hospital is a teaching hospital for the College of Health and Medical Sciences of Haramaya University and serves as a referral hospital for the entire eastern part of our country. The hospital provided health-care services for more than 1850 adult HIV-infected patients in its antiretroviral therapy (ART) clinic [13].

### 2.2. Study Population

The study population comprised of all HIV-positive patients who visited Hiwot Fana Specialized University Hospital ART clinic during the study period. An individual whose is aged below 18 and those who were on antimicrobial treatment for the last two weeks were excluded from the study during data collection.

### 2.3. Sample Size and Sampling Technique

The sample size was determined using a single population proportion formula with the assumption of 10.7% prevalence of UTI [12], 50% for associated factors, 95% confidence level, and a 5% margin of error. The final sample size after the addition of 10% nonresponse was 357. Study participants were selected using a systematic random sampling technique based on daily attendance.

### 2.4. Data and Specimen Collection

Data on sociodemographic characteristics and associated factors were collected using a pretested structured questionnaire. The participant's recent CD4^+^ count was retrieved from their medical record. Single voided clean-catch midstream fresh urine (10 mL) was collected from each participant in a leak-proof and sterilized wide-mouthed screw-capped container. The specimens were kept in a cold box (4°C) and transported within 30 minutes of collection [14] to the Haramaya University College of Health and Medical Sciences Bacteriology laboratory for the analysis.

### 2.5. Isolation and Identification of Bacteria

Bacterial isolation and phenotypic characterizations were performed using the recommended culture and biochemical tests as described by Cheesbrough [15]. A calibrated loop that delivers 0.001 mL of urine was used to inoculate each urine sample onto the Cysteine lactose electrolyte deficient, MacConkey agar, and 5% blood agar (Oxoid Ltd., England) plates. The plates were incubated aerobically at 37°C. After overnight incubation, the phenotypic characterization of bacteria was carried out using colony characteristics and a series of biochemical reactions, including catalase, oxidase, urease, indole, citrate utilization, lysine decarboxylase, glucose, lactose fermentation, gas and H_2_S production, mannitol fermentation, coagulase, and motility tests. A culture growth of ≥10^5^ CFU/mL (colony forming units per milliliter) accompained by symptom was labeled as UTI. Only a single positive culture per patient was included in the analysis [7, 14].

### 2.6. Antimicrobial Susceptibility Testing

Antimicrobial susceptibility test was performed using a Kirby–Bauer disk diffusion method based on the Clinical and Laboratory Standards Institute (CLSI) recommendation [16]. Bacterial inoculum equivalent to 0.5 McFarland standards was prepared by suspending 3–5 freshly grown pure colonies in 5 mL of physiological saline (0.85% NaCl). The suspension was uniformly lawn over the surface of Mueller–Hinton agar (Oxoid Ltd., England) plates using a sterile cotton-tipped applicator. The antimicrobials (Oxoid Ltd., UK) tested were amoxicillin (10 *µ*g), ampicillin (10 *µ*g), cefotaxime (30 *µ*g), cefoxitin (30 *µ*g), ceftazidime (30 *µ*g), ceftriaxone (30 *µ*g), chloramphenicol (30 *µ*g), ciprofloxacin (5 *µ*g), cotrimoxazole (1.25/23.75 *µ*g), gentamycin (10 *µ*g), norfloxacin (10 *µ*g), and tetracycline (30 *µ*g). The disks were placed on the surface of agar plates and incubated at 37°C for 18 to 24 hours. The zone of inhibition was measured to the nearest millimeter and interpreted as sensitive (S), intermediate (I), or resistance (R) based on the interpretative criteria set by the CLSI [16]. Bacterial isolates resistant to two or more antimicrobials belonging to different structural class were classified as multidrug resistant (MDR) [6].

### 2.7. Quality Control

The questionnaire was initially prepared in English, translated into local languages (*Afan Oromo* and *Amharic*) by language experts and back to English by other experts to assure its accuracy. The questionnaire was pretested on 5% HIV-positive patients in the Dilchora Hospital, Dire Dawa, Ethiopia. Data collectors (nurses and medical microbiologists) were trained in questionnaire administration, data collection procedures, culture techniques, bacterial isolation, and antimicrobial susceptibility testing. The new batch culture medium and antimicrobial disks were checked for performance and quality using the American Type Culture Collection (ATCC) reference strains such as *E. coli* (ATCC^®^ 25922), *S. aureus* (ATCC^®^ 25923), and *P. aeruginosa* (ATCC^®^ 27853).

### 2.8. Data Analysis

Data were checked for completeness, coded and entered into the EpiData software (version 2; Odense, Denmark) and exported to the Statistical Package for Social Sciences (version 21, Inc, Chicago, IL) for analysis. Descriptive statistical tools were used to summarize the findings. Bivariate and multivariate logistic regression models were used to predict the relationship between dependent and independent variables. Variables with a *p* value ≤ 0.25 in the bivariate logistic regression were considered in the multivariate logistic regression model. Crude odds ratio (COR) and adjusted odds ratio (AOR) with their 95% confidence interval (CI) were used to determine the significance of the predictors. Variables with a *p* value less than 0.05 in multivariate analysis were taken as significant predictors. The assumption of multivariate logistic regression was checked using Hosmer and Lemeshow goodness-of-fit-test, and a *p* value > 0.05 was considered a good fit.

### 2.9. Ethical Consideration

The study was ethically approved by the Institutional Health Research Ethics Review Committee of the College of Health and Medical Sciences, Haramaya University. Data were collected after informed, voluntary, written, and signed consent secured from each study participant. Participant's information was kept confidential. Positive results were reported to attending physician for appropriate treatment and management.

## 3. Results

### 3.1. Sociodemographic Characteristics

Out of the total (357), 350 HIV-positive adult patients were enrolled in this study, making a response rate of 98%. The majority (69.7%) were females. The mean (±standard deviation) age of the study participants was 35.4 ± (7.4). Most participants had a primary level education (1^st^–8^th^grade) (45.4%), were currently married (61.4%), and were merchants (38.6%). The average monthly income of the participants was less than 46.7 United States Dollar (USD) per month (49.7%) (Table 1).

### 3.2. Prevalence and Associated Factors

Out of the 350 samples, bacteria were isolated from 63 samples giving an overall prevalence of 18% (95% CI: 15.34–22.63). The majority (77.8%) of isolates were Gram-negative bacteria. *E. coli* (38.1%) was the most predominant isolate followed by *K. pneumoniae* (23.8%) and *S. aureus* (11.1%) (Table 2).

The prevalence of UTI was found to be higher in females (73%) followed by the age 35–44 years (55.6%), income less than 46.7 USD (49.2%), and a CD4^+^ count less than 200 cells/mm^3^ (55.6%). Of all considered variables, sex, income, CD4^+^ count/mm^3^, previous history of UTI, history of catheterization, and diabetes were significantly associated with UTI in the bivariate logistic analysis at a *p* value ≤ 0.25. After adjusting for variables tested in the multivariate logistic model, factors such as age 35–44 years (AOR: 4.07; 95% CI: 1.09, 5.10), earning a monthly income of less than 46.7 USD (AOR: 2.76; 95% CI: 1.15, 6.07), and a CD4^+^ count less than 200 cells/mm^3^ (AOR: 2.07; 95% CI: 1.15, 3.73) were significantly associated with a UTI (Table 3).

### 3.3. Antimicrobial Susceptibility Profile

*Escherichia coli* was 95.8% resistant to each of ampicillin, ceftazidime, and cotrimoxazole, 91.7% to amoxicillin, 87.5% to ceftriaxone, and 87.2% to tetracycline. *K. pneumoniae* was 93.3% resistant to cotrimoxazole and 80% to each of amoxicillin and ampicillin (Table 4).

Of Gram-positive bacterial isolates (14), *S. aureus* exhibited high proportion of resistance (85.7%) to each of ampicillin, amoxicillin, and cotrimoxazole. Coagulase-negative *Staphylococcus* isolates were 100% resistant to each of ampicillin and amoxicillin and 80% to chloramphenicol (Table 5).

### 3.4. Multidrug Resistance Pattern

None of the isolates was sensitive or resistant to all the antimicrobials in the testing panel. Multidrug resistance was found in 46% of the isolates. Of these, *E. coli* (37.9%) was the most frequently exhibited MDR followed by *S. aureus* (17.2%) and *K. pneumoniae* (13.8%) (Table 6).

## 4. Discussion

In this study, the prevalence of UTI was 18% (95% CI: 15.34–22.63). Colony counts less than 10^5^ CFU/mL of urine samples were not considered significant to define a UTI. However, in immunocompromised patients, this cannot be ignored. The observation of the high prevalence of UTI in this study may require the need for laboratory investigation as a criterion for the commencement of treatment in HIV-positive patients. This finding was relatively higher than reports in Gondar, Ethiopia (11.9%) [6], and Jimma, Ethiopia (10.7%), [2], but lower than studies conducted in Ebonyi State, Nigeria (93.8%) [1], Tamil Nadu, India (77.5%) [17], and Osogbo, Nigeria (23.5%) [14]. This disparity rate might be attributed to differences in sample size (small sample size might overestimate the proportion), improper collection, and processing of specimens, geographical variation, and socioeconomic conditions.

In the present study, a higher rate of UTI was recorded in the age 35–44 years (55.6%) than 18–24 years with a significant association (AOR: 4.07; 95% CI: 1.09, 5.10). Unlike to this finding, however, relatively higher prevalence was recorded in the age 20–29 years (53.9%) in Irrua, Nigeria (53.9%) [18], and in the age 18–26 years in Gondar, Ethiopia (12.7%) [6]. The higher prevalence of UTI in this study might have attributed to advanced age that reduces the immunity of the individuals (T cells function decreases with increasing age) [4] and personal hygiene.

This study revealed that respondents that earned less than 46.7 USD had a higher prevalence of UTI (49.2%) compared to those who earned greater than 93.5 USD. This pattern was noted to be statistically significant (AOR: 2.76; 95% CI: 1.15, 6.07). Similar findings are documented in studies conducted elsewhere [10, 19]. This may be due to low income that limits the health seeking behavior of an infected individual due to expensive antimicrobials, the high cost of treatment, and unsatisfactory therapeutic options [7, 14].

Urinary tract infections appear to be multifactorial in patients with HIV infections as CD4^+^ level declines [20, 21]. In the present study, individuals who had a CD4^+^ count < 200 cells/mm^3^ were more likely to develop UTI than their counterparts (AOR: 2.07; 95% CI: 1.15, 3.73). Similar findings were reported elsewhere [4, 7, 19, 22]. The results imply that the more immune compromised the patient, the higher the risk of UTI and possibly more vulnerable to other opportunistic infections. The explanation for the inverse relationship of UTI and a CD4^+^ count is unknown; it is probably due to the impaired immunity at a declining CD4^+^ count that makes it easier for bacterial pathogens to adhere to the urinary epithelium.

*Escherichia coli* was found to be the most predominant causative agent of UTI in the present study (38.1%). A similar finding was reported from Gondar, Ethiopia (56.1%) [6], Jimma, Ethiopia (54.3%) [2], and Tertiary Care Hospital, India (41.7%) [23]. This was inconsistent with the finding reported in Ebony State, Nigeria [1] and Tamil Nadu, India [17]. They found *S. aureus* (45.3%) and *P. aeruginosa* (41.9%) as the commonest urinary tract pathogens. The variation in the type of bacteria isolates might be due to differences in sample size, specimen collection technique, sample processing, and personal and environmental hygiene [19]. The preponderance of *E. coli* could be due to the presence of a unique structure that helps these bacteria for attachment to the uroepithelial cells, allowing for multiplication and tissue invasion.

Antimicrobial resistance is a major clinical problem in treating infections caused by different bacterial pathogens and has increased over the years. In the present study, *E. coli* was resistant to ampicillin (95.8%), ceftazidime (95.8%), cotrimoxazole (95.8%), amoxicillin (91.7%), ceftriaxone (87.5%), and tetracycline (87.2%). This was to some extent comparable with the study report in Gondar, Ethiopia [6] and Jimma, Ethiopia [2]. On the other hand, *S. aureus* exhibited 85.7% resistance to each of ampicillin, amoxicillin, and cotrimoxazole. This was comparable with the study done in Gondar, Ethiopia [6]. The resistance to cotrimoxazole may be due to the fact that this drug is widely used for prophylaxis against opportunistic infections associated with HIV [24]. The similarity and differences between reports may be due to the distribution of resistant strains across the country.

Multidrug resistance has serious implications on the health outcome of HIV-infected patients [9, 25]. It is quite alarming to note that almost 46% of the isolates in this study were found to be resistant to two or more antimicrobials. This was higher compared to the finding reported in Mysore, India (28%) [25] but it was lower than a report from Gondar, Ethiopia (95%) [6]. The high rate of resistance seen to the most commonly prescribed antibiotics in this study might be due to easy availability in the community, very cheap in terms of cost and subject to misuse [11]. It could also be due to the use of antibiotics for other nonhuman purposes such as in livestock rearing and animal husbandry activities, which may give force to the growing rate of resistance [1, 25].

The strength of this prospective study was that it evaluated large urine samples for pathogenic bacteria and highlights the emergence of antimicrobial resistance that provides precise scientific data for appropriate treatment, prevention, and control of UTI. However, the study was a single hospital-based study and may not represent all HIV-infected patients. We did not attempt to identify other causative agents (anaerobic bacteria, viruses, and fungus) that would have made a significant contribution to a true prevalence of UTI in HIV-positive patients due to a lack of testing facilities.

## 5. Conclusions

In conclusion, the prevalence of UTI in this study was relatively higher than previous findings in Ethiopia. Age 35–44 years, income less than 46.7 USD, and a CD4^+^ count less than 200 cell/smm^3^ increase the odds of UTI. *E. coli*, *K. pneumoniae*, and *S. aureus* are the major causes of UTI. The isolation of high MDR bacteria highlights the growing challenge of UTIs that is impossible to treat. Health professionals should be aware of regional resistance rates to consider the current empirical antimicrobial therapy for UTI. Measures including health education, continuous monitoring of bacteria, and antimicrobial surveillance are crucial among this group of individuals to mitigate the infection and emergence of antimicrobial resistance. Future studies need to focus on the wider HIV-infected population from several health facilities, exploring a range of causative pathogens and the mechanism of antimicrobial resistance.

## Figures and Tables

**Table 1:** Sociodemographic characteristics of participants at Hiwot Fana Specialized University Hospital, Eastern Ethiopia, 2016.

Characteristics		Total (*n*=350)	
		*n*	%
Sex	Male	106	30.3
	Female	244	69.7

Age (in years)	18–24	12	3.4
	25–34	132	37.7
	35–44	170	48.6
	>44	36	10.3

Educational status	Illiterate	97	27.7
	Grade 1–8	159	45.4
	Grade 9–12	76	21.7
	>12^th^ grade	18	5.1

Current marital status	Unmarried	46	13.1
	In marriage	215	61.4
	Divorced	50	14.3
	Widowed	39	11.3

Occupation	Employee	86	24.6
	Farmer	74	21.1
	Merchant	135	38.6
	Others^*∗*^	55	15.7

Monthly income (in USD)	>93.5	67	19.1
	46.7–93.5	109	31.2
	<46.7	174	49.7

^*∗*^Student, housewife, housemaid, and daily laborer.

**Table 2:** Frequency of bacteria isolates among HIV-positive patients at Hiwot Fana Specialized University Hospital, Eastern Ethiopia, 2016.

Bacterial isolates	*n*	%
*E. coli*	24	38.1
*K. pneumoniae*	15	23.8
*S. aureus*	7	11.1
*P. mirabilis*	6	9.5
Coagulase-negative *Staphylococcus*	5	7.9
*P. aeruginosa*	4	6.4
*Enterococcus* spp.	2	3.2

**Table 3:** Distribution of UTI by associated factors among HIV-positive patients at Hiwot Fana Specialized University Hospital, Eastern Ethiopia, 2016.

Characteristics		Urinary tract infection		Crude OR (95% CI)	Adjusted OR (95% CI)
		Yes, *n* (%)	No, *n* (%)		
Sex	Male	17 (27)	89 (31)	1	
	Female	46 (73)	198 (69)	0.82 [0.45, 1.51]	

Age (in years)	18–24	5 (7.9)	7 (2.4)	1	1
	25–34	17 (27)	115 (40.1)	2.76 [0.83, 9.21]	1.95 [0.55, 6.92]
	35–44	35 (55.6)	135 (47)	4.83 [1.34, 6.96]	4.07 [1.09, 5.10]^*∗∗*^
	>44	6 (9.5)	30 (10.5)	3.57 [0.84, 15.14]	2.63 [0.58, 11.86]

Residence	Urban	42 (67.7)	174 (60.6)	1	
	Rural	21 (32.3)	113 (39.4)	1.29 [0.73, 2.31]	

Educational status	>12^th^ grade	2 (3.2)	16 (5.6)	1	
	Grade 1–8	36 (57.1)	123 (42.9)	0.43 [0.09, 1.95]	
	Grade 9–12	11 (17.7)	65 (22.6)	0.74 [0.15, 3.69]	
	Illiterate	14 (22.6)	83 (28.8)	0.74 [0.15, 3.58]	

Current marital status	Unmarried	11 (17.5)	35 (12.2)	1	
	Married	37 (58.7)	178 (62)	1.51 [0.70, 3.25]	
	Divorced	8 (12.7)	42 (14.6)	1.65 [0.59, 4.55]	
	Widowed	7 (11.1)	32 (11.1)	1.44 [0.49, 4.16]	

Occupation	Employed	17 (27)	69 (24)	1	
	Farmer	10 (15.9)	64 (22.3)	1.58 [0.67, 3.69]	
	Merchant	24 (38.1)	111 (38.7)	1.14 [0.57, 2.27]	
	Others^*∗*^	12 (19)	43 (15)	0.88 [0.38, 2.03]	

Average monthly income (in USD)	>93.5	14 (22.2)	53 (18.5)	1	1
	46.7–93.5	18 (28.6)	91 (31.7)	0.96 [0.36, 1.42]	0.76 [0.36, 1.56]
	<46.7	31 (49.2)	143 (49.8)	2.28 [1.07, 4.92]	2.76 [1.15, 6.07]^*∗∗*^

Current CD4^+^ count (cells/mm^3^)	≥200	28 (44.4)	89 (31)	1	1
	<200	35 (55.6)	198 (69)	1.78 [1.02. 3.10]	2.07 [1.15, 3.73]^*∗∗*^

ART status	Nonusers	20 (31.7)	76 (26.5)	1	
	Users	43 (68.3)	211 (73.5)	1.29 [0.72, 2.33]	

Use of cotrimoxazole as prophylaxis	No	25 (39.7)	123 (42.9)	1	
	Yes	38 (60.3)	164 (57.1)	0.88 [0.50, 1.53]	

Use of drugs without prescription	Yes	8 (12.7)	24 (8.4)	1	
	No	55 (87.3)	263 (91.6)	1.59 [0.68, 3.73]	

Previous history of UTI	No	49 (77.8)	232 (80.8)	1	
	Yes	14 (22.2)	55 (19.2)	0.83 [0.43, 1.61]	

History of catheterization	No	59 (93.7)	270 (94.1)	1	
	Yes	5 (5.9)	16 (5.9)	0.69 [0.24, 1.94]	

Diabetes	No	58 (92.1)	274 (95.5)	1	
	Yes	5 (7.9)	13 (4.5)	0.55 [0.19, 1.60]	

Sexual activities in the last 3 months	No	18 (28.6)	67 (23.3)	1	
	Yes	45 (71.4)	220 (76.7)	1.31 [0.71, 2.42]	

Frequency of sexual activity per week	No	15 (23.8)	59 (20.6)	1	
	1‐2 times	20 (31.7)	153 (53.3)	0. 68 [0.33, 1.39]	
	>2 times	28 (44.4)	75 (26.1)	1.95 [0.93, 4.05]	

^*∗*^Student, housewife, housemaid, and daily laborer; ^*∗∗*^*p* < 0.05; OR: odds ratio; CI: confidence interval.

**Table 4:** Antimicrobial susceptibility pattern of Gram-negative bacterial isolates among HIV-positive patients at Hiwot Fana Specialized University Hospital, Eastern Ethiopia, 2016.

Bacterial isolates, *n*		Pattern	Antimicrobial susceptibility pattern, *n* (%)									
			AM	AML	CTX	CAZ	CRO	CIP	GN	NOR	TE	COT
*E. coli*	24	S	0 (0)	1 (4.2)	17 (70.8)	0 (0)	2 (8.3)	17 (70.8)	19 (79.2)	20 (83.3)	1 (4.2)	1 (4.2)
		I	1 (4.2)	1 (4.2)	0 (0)	1 (4.2)	1 (4.2)	2 (8.3)	2 (8.3)	1 (4.2)	2 (8.3)	0 (0)
		R	23 (95.8)	22 (91.7)	7 (29.2)	23 (95.8)	21 (87.5)	3 (20.5)	3 (12.2)	3 (12.5)	21 (87.2)	23 (95.8)

*K. pneumoniae*	15	S	3 (20)	2 (13.3)	11 (73.3)	4 (26.7)	9 (60)	12 (80)	12 (80)	12 (80)	9 (60)	1 (6.7)
		I	0 (0)	1 (6.7)	0 (0)	1 (6.7)	2 (13.3)	0 (0)	1 (6.7)	0 (0)	0 (0)	0 (0)
		R	12 (80)	12 (80)	4 (26.7)	10 (66.7)	4 (26.7)	3 (20)	2 (13.3)	3 (20)	6 (40)	12 (93.3)

*P. mirabilis*	6	S	3 (50)	2 (33.3)	4 (66.7)	6 (100)	6 (100)	5 (83.3)	5 (83.3)	6 (100)	1 (16.7)	2 (33.3)
		I	1 (16.7)	0 (0)	0 (0)	0 (0)	0 (0)	0 (0)	0 (0)	0 (0)	0 (0)	0 (0) 0
		R	2 (33.3)	4 (66.7)	2 (33.3)	0 (0)	0 (0)	1 (16.7)	1 (16.7)	0 (0)	5 (83.3)	4 (66.7)

*P. aeruginosa*	4	S	0 (0)	0 (0)	—	1 (25)	—	3 (75)	0 (0)	1 (25)	0 (0)	0 (0)
		I	0 (0)	0 (0)	—	0 (0)	—	0 (0)	0 (0)	0 (0)	0 (00)	0 (0)
		R	4 (100)	4 (100)	—	3 (75)	—	1 (25)	4 (100)	3 (75)	4 (100)	4 (100)

Total, *n* (%)	49	S	8 (16.2)	5 (10.2)	32 (65.3)	11 (22.4)	17 (34.7)	35 (74.1)	36 (76.5)	37 (75.5)	11 (22.4)	4 (8.2)
		I	2 (4.1)	2 (4.1)	0 (0)	2 (4.1)	3 (6.1)	2 (4.1)	3 (6.1)	1 (2)	2 (4.1)	0 (0)
		R	39 (79.6)	42 (85.4)	13 (26.5)	36 (73.5)	25 (51)	12 (24.5)	10 (20.4)	9 (18.7)	36 (73.5)	45 (91.8)

S: sensitive; I: intermediate sensitive; R: resistant; AM: ampicillin; AML: amoxicillin; CTX: cefotaxime; CAZ: ceftazidime; CRO: ceftriaxone; CIP: ciprofloxacin; GN: gentamicin; NOR: norfloxacin; TE: tetracycline; COT: cotrimoxazole.

**Table 5:** Antimicrobial susceptibility pattern of Gram-positive bacterial isolates to antimicrobial agents among HIV-positive patients at Hiwot Fana Specialized University Hospital, Eastern Ethiopia, 2016.

Bacterial isolates, *n*		Pattern	Antimicrobial susceptibility pattern, *n* (%)								
			AM	AML	C	FOX	CIP	GN	NOR	TE	COT
*S. aureus*	7	S	1 (14.3)	1 (14.3)	4 (57.1)	3 (42.9)	4 (57.1)	3 (42.9)	4 (57.1)	2 (28.6)	1 (14.3)
		I	0 (0)	0 (0)	0 (0)	0 (0)	0 (0)	0 (0)	0 (0)	1 (14.3)	0 (0)
		R	6 (85.7)	6 (85.7)	3 (42.9)	4 (57.1)	3 (42.9)	4 (57.1)	3 (42.9)	4 (57.1)	6 (85.7)

CoNS	5	S	0 (0)	0 (0)	1 (20)	5 (100)	3 (60)	3 (60)	3 (60)	1 (20)	1 (20)
		I	0 (0)	0 (0)	0 (0)	0 (0)	0 (0)	0 (0)	0 (0)	2 (40)	2 (40)
		R	5 (100)	5 (100)	4 (80)	0 (0)	2 (40)	2 (40)	2 (40)	2 (40)	2 (40)

*Enterococcus* spp.	2	S	0 (0)	0 (0)	2 (100)	1 (50)	1 (50)	2 (50)	2 (100)	1 (50)	0 (0)
		I	0 (0)	0 (0)	0 (0)	1 (50)	0 (0)	0 (0)	0 (0)	1 (50)	0 (0)
		R	2 (100)	2 (100)	0 (0)	0 (0)	1 (50)	0 (0)	0 (0)	0 (0)	2 (100)

Total, *n* (%)	14	S	11 (78.6)	1 (7.1)	4 (28.6)	9 (64.3)	8 (57.1)	9 (64.3)	9 (64.3)	2 (14.3)	2 (14.3)
		I	0 (0)	0 (0)	0 (0)	1 (7.1)	0 (0)	0 (0)	0 (0)	4 (28.6)	2 (14.3)
		R	3 (21.4)	13 (92.9)	10 (71.4)	4 (28.6)	6 (48.6)	5 (35.7)	5 (35.7)	8 (57.1)	10 (74.4)

S: sensitive; I: intermediate sensitive; R: resistant; CoNS: coagulase-negative *Staphylococcus*; AM: ampicillin; AML: amoxicillin; C: chloramphenicol; FOX: cefoxitin; CIP: ciprofloxacin; GN: gentamicin; NOR: norfloxacin; TE: tetracycline; COT: cotrimoxazole.

**Table 6:** Pattern of multidrug-resistant bacteria among HIV-positive patients at Hiwot Fana Specialized University Hospital, Eastern Ethiopia, 2016.

Antimicrobials, *n* (%)		MDR bacterial isolates, *n* (%)						
		*E. coli*	*K. pneumoniae*	*P. mirabilis*	*P. aeruginosa*	*S. aureus*	*CoNS*	*Enterococcus* spp.
AM, AML	8 (17.4)	3 (37.5)	1 (12.5)	0 (0)	0 (0)	2 (25)	2 (25)	0 (0)
AML, COT	3 (6.5)	2 (18.2)	0 (0)	0 (0)	1 (33.3)	0 (0)	0 (0)	0 (0)
TE, COT	3 (6.5)	2 (66.7)	0 (0)	1 (33.3)	0 (0)	0 (0)	0 (0)	0 (0)
AML, AML, COT	5 (10.9)	1 (20)	1 (20)	1 (20)	1 (20)	0 (0)	0 (0)	1 (20)
AM, AML, NOR	3 (6.5)	0 (0)	0 (0)	0 (0)	1 (33.3)	1 (33.3)	1 (33.3)	0 (0)
AM, AML, CAZ	2 (5.3)	1 (50)	1 (50)	0 (0)	0 (0)	0 (0)	0 (0)	0 (0)
AML, TE, COT	3 (6.5)	1 (33.3)	0 (0)	0 (0)	0 (0)	2 (66.7)	0 (0)	0 (0)
AML, CIP, GN	2 (5.3)	1 (50)	1 (50)	0 (0)	0 (0)	0 (0)	0 (0)	0 (0)
MDR, *n* (%)	29 (46)	11 (37.9)	4 (13.8)	2 (6.9)	3 (10.3)	5 (17.2)	3 (10.3)	1 (3.4)

AM: ampicillin; AML: amoxicillin; CAZ: ceftazidime; CRO: ceftriaxone; CIP: ciprofloxacin; GN: gentamicin; NOR: norfloxacin; TE: tetracycline; COT: cotrimoxazole.

## Data Availability

The SPSS data used to support the findings of this study are included within the article.
